# Correction for Kommedal et al., “Microbiological diagnosis of pleural infections: a comparative evaluation of a novel syndromic real-time PCR panel”

**DOI:** 10.1128/spectrum.02938-24

**Published:** 2025-01-13

**Authors:** Øyvind Kommedal, Tomas Mikal Eagan, Øystein Fløtten, Truls Michael Leegaard, William Siljan, Hilde Fardal, Bjørnar Bø, Fredrik Grøvan, Kjersti Wik Larssen, Arne Kildahl-Andersen, Reidar Hjetland, Rune Tilseth, Sølvi Kristine Øyen Hareide, Marit Tellevik, Ruben Dyrhovden

## AUTHOR CORRECTION

Volume 12, no. 6, e03510-23, 2024, https://doi.org/10.1128/spectrum.03510-23. Page 4, Table 1, MP4 rows: “*nusG*” should read “*recQ*,” “TGGGCTTTATTGGTGGTA” should read “CTGACTGCCAATATTCCG,” “GKTTACGCGCACTTCTTC” should read “GCATGAATAACCARAAGATTCG,” “CGCCAATTAGTAAYCGTGAAGCAGAT” should read “TGCCGATACCGTACACGCTAAG,” and “143” should read “93.”

