# Supplementary material for: Microbiological diagnosis of pleural infections: a comparative evaluation of a novel syndromic real-time PCR panel
Source: Microbiol Spectr. 2024 Apr 24;12(6):e03510-23. doi: 10.1128/spectrum.03510-23 (PMC11237507; doi:10.1128/spectrum.03510-23)
Supplement: Document S1 — PCR target details. [file spectrum.03510-23-s0001.docx]

Supplementary document S1: Target considerations and oligonucleotide design

Primers and probes were designed using the OligoArchitect online software tool (Sigma-Aldrich, St. Louis, Missouri, US) for primer and probe designs. Candidate primer/probe target-coverage and specificity was evaluated in-silico using NCBI GenBank/BLAST. All primers and probes were ordered from TibMolBiol (Berlin, Germany).

The taxonomy of the oral microbiota is continuously evolving. Up to recently, *F. nucleatum* was divided into four subspecies, which have now been elevated into separate species (*Fusobacterium animalis*, *F. nucleatum*, *Fusobacterium polymorphum* and *Fusobacterium vincentii*). In addition, another two closely related species have been described (*Fusobacterium watanabei* and *Fusobacterium hwasookii*) together with the provisionally named species “*Fusobacterium sp. HMT-203*”. Since little is known about eventual differences in the pathogenic potential of these species and 16S TNGS discriminates poorly, we designed the Fnucl-PCR to cover all of them. We also included a PCR for *Fusobacterium necrophorum* and *Fusobacterium gonidiaformans* since we assume that these can have a similar role as the *Fusobacterium nucleatum* group.

Similar considerations were made for the Aaphr-PCR that targets both *A. aphrophilus* and the novel closely related species *Aggregatibacter kilianii* and the Pmicr-PCR that target both *Parvimonas micra* and the closely related oral species provisionally named *Parvimonas sp. HMT-110*. As *P. micra* seems to always be detected together with either *F. nucleatum* or *A. aphrophilus* the inclusion of a *P. micra* PCR might not be necessary. We decided to keep it, since it is also the third most common CAPI pathogen.

Although we have not observed mono-microbial empyema with *Streptococcus constellatus* and it always appeared together with either *F. nucleatum* or *S. intermedius* in the present study, we have previously reported it in a mono-bacterial brain-abscess [(19)](https://app.readcube.com/library/214adaea-5dc4-4061-8108-bb5d24e4525f/all?uuid=45562677118282613&item_ids=214adaea-5dc4-4061-8108-bb5d24e4525f:11eec593-521a-482a-8399-d6c229f5ac99). We therefore chose to cover it in the Sintcon-PCR together with *S. intermedius*.

The PCRs for the eleven targets were combined into four duplex and one triplex PCRs (Multiplex PCRs 1-5, Table 1). To prevent reduced sensitivity due to internal competition, we avoided combining PCRs for bacteria that are frequently co-identified in the same infections. We still found it acceptable to combine the Fnucl, Fnecgon and Pmicr PCRs since they are all indicators of a potential polymicrobial/anaerobic infection.
