# Supplementary material for: Microbiological diagnosis of pleural infections: a comparative evaluation of a novel syndromic real-time PCR panel
Source: Microbiol Spectr. 2024 Apr 24;12(6):e03510-23. doi: 10.1128/spectrum.03510-23 (PMC11237507; doi:10.1128/spectrum.03510-23)
Supplement: Supplemental Tables — Tables S1 to S6. [file spectrum.03510-23-s0002.docx]

Supplementary Table S1: Alternative PCRs for *P. micra* and *S. aureus*

| **PCR** | **Gene^a^** | **P/Pb** | **Cons. (µM)** | **Sequence (5’ – 3’)** | **Amplicon size** | **Fl/Q** |
| --- | --- | --- | --- | --- | --- | --- |
| *S. aureus* | *nusG* | F | 0.4 | ACTGGTTTTGTAGGTTCTG | 125 bp | FAM/BHQ1 |
|  |  | R | 0.4 | CCAACTTCGAGTTCAACA |  |  |
|  |  | Pb | 0.2 | AATCCRTTGTTACCAGAAGAAGTTCGC |  |  |
| *P. micra* | *recA* | F | 0.4 | AGGAGCCATTTTATTCTTAACA | 108 bp | FAM/BHQ1 |
|  |  | R | 0.4 | GCAAGTGTTAGAATTGATATAAGG |  |  |
|  |  | Pb | 0.2 | TTGCTCCATAAGGTTCTCCATCTCC |  |  |

*a = nusG*: Transcription regulation; *recA*: Recombinase A

Supplementary Table S2: Technical performance of the individual PCRs in their respective multiplex-PCRs

| MP | PCR^a^ | Estimated efficacy | Estimated sensitivity^b^ | Expected sensitivity^c^ |
| --- | --- | --- | --- | --- |
| MP1 | Fnecgon | 100% | 0.3 | 1-10 |
|  | Fnucl | 95% | 0.4 | 1-10 |
|  | Pmicr | 100% | 0.7 | 1-10 |
| MP2 | Spyog | 102% | 2.8 | 1-10 |
|  | Sintcon | 102% | 0.3 | 1-10 |
| MP3 | Spneu | 98% | 0.2 | 1-10 |
|  | Hinfl | 102% | 3.3 | 1-10 |
| MP4 | Aaphr | 95% | 0.2 | 1-10 |
|  | Saure | 100% | 1.6 | 1-10 |
| MP5 | Paeru | 103% | 1.0 | 1-10 (10-100)^d^ |
|  | Ebact | 104% | 1.0 | 1-10 (10-100)^d^ |

MP = Multiplex PCR

a = Fnecgon-PCR targets *F. necrophorum* and *F. gonidiaformans*. Fnucl-PCR targets *F. nucleatum* and the closely related species *F. vincentii*, *F. animalis*, *F. polymorphum*, “*Fusobacterium sp. HMT-203*”, *F. hwasookii*, *F. periodonticum*, “*F. pseudoperiodonticum*” and *F. watanabei*. Pmicr-PCR targets *P. micra* and “*Parvimonas sp. HMT-110*”. Spyog-PCR targets *S. pyogenes*. Sintcon-PCR targets *S. intermedius* and *S. constellatus*. Aaphr-PCR targets *A. aphrophilus* and *A. kilianii*. Saure-PCR targets S. aureus. Paeru-PCR targets *P. aeruginosa*. Ebact-PCR targets the genera *Citrobacter*, *Enterobacter*, *Escherichia*, *Hafnia*, *Klebsiella*, *Raoultella*, *Salmonella* and *Serratia*.

b = Estimated number of copies per reaction. Based on 10-fold dilution series of control strains. Last dilution step where all three replicates were detected.

c = target copies per reaction. Expected real-life sensitivity.

d = reduced sensitivity due to cutoff at PCR cycle 33.

Supplementary Table S3: Result discrepancies between the CAPI-PCR and 16S TNGS

| ID | Concordant findings | PCR  Ct-value | Additional PCR findings | PCR  Ct-value | Confirmed alternative PCR | Confirmed by culture | Supported by NGS below cutoff^a^ |
| --- | --- | --- | --- | --- | --- | --- | --- |
| PE01 | *E. cloacae* | 22 | *F. nucleatum* | 36 | NA | NO | **YES** |
|  |  |  | *P. micra* | 37 | **YES** | NO | NO |
| PE11 | *F. nucleatum* | 26 | *S. aureus* | 36 | **YES** | **YES** | NO |
|  | *P. micra* | 26 |  |  |  |  |  |
|  | *S. intermedius* | 22 |  |  |  |  |  |
| PE34 | *F. nucleatum* | 33 | *P. micra* | 36 | **YES** | NO | NO |
|  | *S. intermedius* | 33 |  |  |  |  |  |
| PE38 | *F. nucleatum* | 34 | *P. micra* | 36 | **YES** | NO | **YES** |
|  | *S. intermedius* | 34 |  |  |  |  |  |
| PE42 | *F. nucleatum* | 33 | *P. micra* | 35 | **YES** | NO | NO |
|  | *S. intermedius* | 30 |  |  |  |  |  |
| PE52 | *F. nucleatum* | 31 | *P. micra* | 35 | **YES** | NO | NO |
| PE73 | *F. nucleatum* | 32 | *P. micra* | 36 | **YES** | NO | **YES** |
|  | *S. intermedius* | 32 |  |  |  |  |  |
| E07 | *F. nucleatum* | 20 | *H. influenzae* | 34 | NA | NO | **YES** |
|  | *S. intermedius* | 23 |  |  |  |  |  |
| E17 | *F. nucleatum* | 35 | *S. intermedius* | 37 | NA | **YES (BC)^b^** | **YES** |
|  | *P. micra* | 36 |  |  |  |  |  |
| E18 | *F. nucleatum* | 25 | *S. aureus* | 37 | NRE | **YES (BC)^b^** | **YES** |
|  | *P. micra* | 25 |  |  |  |  |  |
|  | *S. intermedius* | 31 |  |  |  |  |  |
| E21 | *F. nucleatum* | 26 | *P. micra* | 36 | **YES** | NO | NO |
|  | *S. intermedius* | 19 | *S. pneumoniae* | 38 | NA | NO | **YES** |

ID = sample identity in references (6) (PExx) and (4) (Exx). NA = no alternative PCR available; NRE = no residual eluate available; BC = blood culture

a = present in NGS-result, but below cutoff/removed by filtering

b = pleural fluid inoculated in blood culture bottle bedside

Supplementary Table S4: Samples where PCR detected all species present

| Sample composition | Number of samples |
| --- | --- |
| *S. intermedius* | 32 |
| *S. pneumoniae* | 11 |
| *F. nucleatum* | 5 |
| *S. aureus* | 3 |
| *S. pyogenes* | 2 |
| *E. coli* | 2 |
| *A. aphrophilus* | 1 |
| *P. aeruginosa* | 1 |
| *S. intermedius*, *F. nucleatum* | 5 |
| *S. intermedius, F. nucleatum, P. micra* | 3 |
| *F. nucleatum*, *P. micra* | 1 |
| *S. intermedius*, *F. nucleatum*, *P. micra, A. aphrophilus* | 1 |
| *S. aureus*, *S. pyogenes* | 1 |
| *E. cloacae*, *F. nucleatum*, *P. micra* | 1 |
| TOTAL number of samples | 69 |

Supplementary Table S5: PCR non-target species in CAPI and their prevalences in this study

|  | Species | detections (n) | Prevalence % |
| --- | --- | --- | --- |
| 1 | *Eubacterium brachy* | 18 | 16.5 % |
| 2 | *Prevotella oris* | 11 | 10.1 % |
| 3 | *Porphyromonas endodontalis* | 10 | 9.2 % |
| 4 | *Campylobacter gracilis* | 8 | 7.3 % |
| 5 | *Campylobacter rectus* | 8 | 7.3 % |
| 6 | *Dialister pneumosintes* | 7 | 6.4 % |
| 7 | *Prevotella pleuritidis* | 6 | 5.5 % |
| 17 | *Eikenella corrodens/exigua* | 6 | 5.5 % |
| 8 | *Filifactor alocis* | 5 | 4.6 % |
| 9 | *Peptostreptococcaceae [G-4] sp. HMT-369* | 5 | 4.6 % |
| 10 | *Peptostreptococcus stomatis* | 5 | 4.6 % |
| 11 | *Prevotella conceptionensis* | 5 | 4.6 % |
| 12 | *Tannerella forsythia* | 5 | 4.6 % |
| 13 | *Treponema lecithinolyticum* | 5 | 4.6 % |
| 14 | *Alloprevotella tannerae* | 4 | 3.7 % |
| 15 | *Catonella morbi* | 4 | 3.7 % |
| 16 | *Dialister invisus* | 4 | 3.7 % |
| 18 | *Lancefieldella rimae* | 4 | 3.7 % |
| 19 | *Mycoplasma faucium* | 4 | 3.7 % |
| 20 | *Slackia exigua* | 4 | 3.7 % |
| 21 | *Alloprevotella rava* | 3 | 2.8 % |
| 22 | *Eubacterium nodatum* | 3 | 2.8 % |
| 23 | *Mycoplasma salivarium* | 3 | 2.8 % |
| 24 | *Porphyromonas asaccharolytica* | 3 | 2.8 % |
| 25 | *Prevotella buccae* | 3 | 2.8 % |
| 26 | *Prevotella intermedia* | 3 | 2.8 % |
| 27 | *Actinomyces meyeri* | 2 | 1.8 % |
| 28 | *Aggregatibacter aphrophilus* | 2 | 1.8 % |
| 29 | *Bacteroidales [G-2] sp. HMT-274* | 2 | 1.8 % |
| 30 | *Bacteroidetes [G-3] sp. HMT-365* | 2 | 1.8 % |
| 31 | *Bacteroidetes [G-7] sp. HMT-911* | 2 | 1.8 % |
| 32 | *Eubacterium infirmum* | 2 | 1.8 % |
| 33 | *Gemella bergeriae* | 2 | 1.8 % |
| 34 | *Gemella morbillorum* | 2 | 1.8 % |
| 35 | *Mogibacterium timidum* | 2 | 1.8 % |
| 36 | *Peptoanaerobacter stomatis* | 2 | 1.8 % |
| 37 | *Peptococcus sp. HMT-167* | 2 | 1.8 % |
| 38 | *Porphyromonas gingivalis* | 2 | 1.8 % |
| 39 | *Prevotella baroniae* | 2 | 1.8 % |
| 40 | *Prevotella nigrescens* | 2 | 1.8 % |
| 41 | *Prevotella spE734* | 2 | 1.8 % |
| 42 | *Schaalia meyeri* | 2 | 1.8 % |
| 43 | *Streptococcus mitis* | 2 | 1.8 % |
| 44 | *Treponema maltophilum* | 2 | 1.8 % |
| 45 | *Treponema denticola* | 2 | 1.8 % |
| 46 | *Actinomyces funkei* | 1 | 0.9 % |
| 47 | *Actinomyces urinae* | 1 | 0.9 % |
| 48 | *Alloprevotella sp. HMT-308* | 1 | 0.9 % |
| 49 | *Anaerococcus obesiensis* | 1 | 0.9 % |
| 50 | *Anaerococcus vaginalis* | 1 | 0.9 % |
| 51 | *Atopobium deltae* | 1 | 0.9 % |
| 52 | *Bulleidia extructa* | 1 | 0.9 % |
| 53 | *Campylobacter ureolyticus* | 1 | 0.9 % |
| 54 | *Capnocytophaga sp. HMT-338* | 1 | 0.9 % |
| 55 | *Catonella sp. HMT-451* | 1 | 0.9 % |
| 56 | *Clostridiales [F1][G1] sp. HMT-093* | 1 | 0.9 % |
| 57 | *Clostridium perfringens* | 1 | 0.9 % |
| 58 | *Colibacter massiliensis* | 1 | 0.9 % |
| 59 | *Eggerthia catenaformis* | 1 | 0.9 % |
| 60 | *Erysipelotrichaceae [G1] sp. HMT-905* | 1 | 0.9 % |
| 61 | *Eubacterium saphenum* | 1 | 0.9 % |
| 62 | *Eubacterium yurii* | 1 | 0.9 % |
| 63 | *Fenollaria massiliensis* | 1 | 0.9 % |
| 64 | *Fretibacterium sp. HMT-359* | 1 | 0.9 % |
| 65 | *Gemella asaccharolytica* | 1 | 0.9 % |
| 66 | *Gleimia europaea* | 1 | 0.9 % |
| 67 | *Lachnoanaerobaculum gingivalis* | 1 | 0.9 % |
| 68 | *Lachnospiraceae [G7] sp. HMT-086* | 1 | 0.9 % |
| 69 | *Lawsonella clevelandensis* | 1 | 0.9 % |
| 70 | *Leptotrichia amnionii* | 1 | 0.9 % |
| 71 | *Moryella indoligenes* | 1 | 0.9 % |
| 72 | *Olsenella uli* | 1 | 0.9 % |
| 73 | *Oribacterium sp. HMT-102* | 1 | 0.9 % |
| 74 | *Peptococcus niger* | 1 | 0.9 % |
| 75 | *Peptoniphilus lacrimalis* | 1 | 0.9 % |
| 76 | *Peptostreptococcus anaerobius* | 1 | 0.9 % |
| 77 | *Porphyromonas somerae* | 1 | 0.9 % |
| 78 | *Prevotella denticola* | 1 | 0.9 % |
| 79 | *Prevotella melaninogenica* | 1 | 0.9 % |
| 80 | *Prevotella bergensis* | 1 | 0.9 % |
| 81 | *Prevotella dentalis* | 1 | 0.9 % |
| 82 | *Prevotella sp. HMT-304* | 1 | 0.9 % |
| 83 | *Prevotella sp. HMT-472* | 1 | 0.9 % |
| 84 | *Prevotella sp. HMT-526* | 1 | 0.9 % |
| 85 | *Prevotella loescheii* | 1 | 0.9 % |
| 86 | *Prevotella sp. 6021087986* | 1 | 0.9 % |
| 87 | *Prevotella timonensis* | 1 | 0.9 % |
| 88 | Pseudoramibacter alactolyticus | 1 | 0.9 % |
| 89 | Schaalia turicensis | 1 | 0.9 % |
| 90 | Solobacterium moorei | 1 | 0.9 % |
| 91 | Staphylococcus saccharolyticus | 1 | 0.9 % |
| 92 | Staphylococcus warneri | 1 | 0.9 % |
| 93 | Streptococcus dysgalactiae | 1 | 0.9 % |
| 94 | Streptococcus lactarius | 1 | 0.9 % |
| 95 | Treponema sp. HMT-237 | 1 | 0.9 % |
| 96 | Treponema pectinovorum | 1 | 0.9 % |
| 97 | Treponema socranskii | 1 | 0.9 % |
|  |  | 236 |  |

Supplementary Table S6: Diagnostic performance of CAPI-PCR on 109 CAPIs and 11 negative controls.

| **a) Presence of bacteria in fluid (diagnostic sensitivity and specificity)** | | | | | | | | |
| --- | --- | --- | --- | --- | --- | --- | --- | --- |
|  |  | Composite reference standard | |  |  |  |  |  |
|  |  | Yes | No | Total |  |  |  |  |
| CAPI-PCR | Yes | 107 | 0 | 107 |  | Sensitivity (95% CI) | 0,98 | (0,94-1,00) |
|  | No | 2 | 11 | 13 |  | Spesificity (95% CI) | 1,00 | (0,72-1,00) |
|  | Total | 109 | 11 | 120 |  |  |  |  |

| **b) Detection of bacteria targeted by PCR** | | | | | | | | |
| --- | --- | --- | --- | --- | --- | --- | --- | --- |
|  |  | All bacterial detections by composite reference standard | |  | | | |  |
|  |  | Included in CAPI-PCR | Not included in CAPI-PCR | Total |  |  |  |  |
| CAPI-PCR | Yes | 184 | 0 | 184 |  | Sensitivity (95% CI) | 0,99 | (0,97-1,00) |
|  | No | 1 | 236 | 237 |  | Specificity (95% CI) | 1,00 | (0,98-1,00) |
|  | Total | 185 | 236 | 421 |  |  |  |  |
|  |  |  |  |  |  |  |  |  |

| **c) Accuracy of each PCR included in the CAPI-PCR panel** | | | | | | | | |
| --- | --- | --- | --- | --- | --- | --- | --- | --- |
|  |  |  |  |  |  |  |  |  |
| **Fusobacterium nucleatum group/gonidiaformans/necrophorum PCR** | | | | | | | | |
|  |  | Composite reference standard | |  |  |  |  |  |
|  |  | Positive | Negative | Total |  |  |  |  |
| CAPI-PCR | Positive | 55 | 0 | 55 |  | Sensitivity (95% CI) | 1,00 | (0,93-1,00) |
|  | Negative | 0 | 65 | 65 |  | Spesificity (95% CI) | 1,00 | (0,95-1,00) |
|  | Total | 55 | 65 | 120 |  |  |  |  |
|  |  |  |  |  |  |  |  |  |
| **Parvimonas micra PCR** | | | | | | | | |
|  |  | Composite reference standard | |  |  |  |  |  |
|  |  | Positive | Negative | Total |  |  |  |  |
| CAPI-PCR | Positive | 33 | 0 | 33 |  | Sensitivity (95% CI) | 1,00 | (0,89-1,00) |
|  | Negative | 0 | 87 | 87 |  | Spesificity (95% CI) | 1,00 | (0,96-1,00) |
|  | Total | 33 | 87 | 120 |  |  |  |  |
|  |  |  |  |  |  |  |  |  |
| **Aggreagatibacter aprophilus/kilianii PCR** | | | | | | | | |
|  |  | Composite reference standard | |  |  |  |  |  |
|  |  | Positive | Negative | Total |  |  |  |  |
| CAPI-PCR | Positive | 3 | 0 | 3 |  | Sensitivity (95% CI) | 1,00 | (0,29-1,00) |
|  | Negative | 0 | 117 | 117 |  | Spesificity (95% CI) | 1,00 | (0,97-1,00) |
|  | Total | 3 | 117 | 120 |  |  |  |  |
|  |  |  |  |  |  |  |  |  |
| **Streptococcus intermedius/constellatus PCR** | | | | | | | | |
|  |  | Composite reference standard | |  |  |  |  |  |
|  |  | Positive | Negative | Total |  |  |  |  |
| CAPI-PCR | Positive | 67 | 0 | 67 |  | Sensitivity (95% CI) | 1,00 | (0,95-1,00) |
|  | Negative | 0 | 53 | 53 |  | Spesificity (95% CI) | 1,00 | (0,93-1,00) |
|  | Total | 67 | 53 | 120 |  |  |  |  |
|  |  |  |  |  |  |  |  |  |
| **Staphyloccus aureus PCR** | | | | | | | | |
|  |  | Composite reference standard | |  |  |  |  |  |
|  |  | Positive | Negative | Total |  |  |  |  |
| CAPI-PCR | Positive | 6 | 0 | 6 |  | Sensitivity (95% CI) | 1,00 | (0,54-1,00) |
|  | Negative | 0 | 114 | 114 |  | Spesificity (95% CI) | 1,00 | (0,97-1,00) |
|  | Total | 6 | 114 | 120 |  |  |  |  |
|  |  |  |  |  |  |  |  |  |
| **Streptococcus pyogenes PCR** | | | | | | | | |
|  |  | Composite reference standard | |  |  |  |  |  |
|  |  | Positive | Negative | Total |  |  |  |  |
| CAPI-PCR | Positive | 3 | 0 | 3 |  | Sensitivity (95% CI) | 1,00 | (0,29-1,00) |
|  | Negative | 0 | 117 | 117 |  | Spesificity (95% CI) | 1,00 | (0,97-1,00) |
|  | Total | 3 | 117 | 120 |  |  |  |  |
|  |  |  |  |  |  |  |  |  |
| **Pseudomoas aeruginosa PCR** | | | | | | | | |
|  |  | Composite reference standard | |  |  |  |  |  |
|  |  | Positive | Negative | Total |  |  |  |  |
| CAPI-PCR | Positive | 1 | 0 | 1 |  | Sensitivity (95% CI) | 1,00 | (0,03-1,00) |
|  | Negative | 0 | 119 | 119 |  | Spesificity (95% CI) | 1,00 | (0,97-1,00) |
|  | Total | 1 | 119 | 120 |  |  |  |  |
|  |  |  |  |  |  |  |  |  |
|  |  |  |  |  |  |  |  |  |
| **Streptococcus pneumoniae PCR** | | | | | | | | |
|  |  | Composite reference standard | |  |  |  |  |  |
|  |  | Positive | Negative | Total |  |  |  |  |
| CAPI-PCR | Positive | 12 | 0 | 12 |  | Sensitivity (95% CI) | 1,00 | (0,73-1,00) |
|  | Negative | 0 | 108 | 108 |  | Spesificity (95% CI) | 1,00 | (0,97-1,00) |
|  | Total | 12 | 108 | 120 |  |  |  |  |
|  |  |  |  |  |  |  |  |  |
| **Haemophilus influenzae PCR** | | | | | | | | |
|  |  | Composite reference standard | |  |  |  |  |  |
|  |  | Positive | Negative | Total |  |  |  |  |
| CAPI-PCR | Positive | 1 | 0 | 1 |  | Sensitivity (95% CI) | 1,00 | (0,03-1,00) |
|  | Negative | 0 | 119 | 119 |  | Spesificity (95% CI) | 1,00 | (0,97-1,00) |
|  | Total | 1 | 119 | 120 |  |  |  |  |
|  |  |  |  |  |  |  |  |  |
| **Enterobacterales PCR** | | | | | | | | |
|  |  | Composite reference standard | |  |  |  |  |  |
|  |  | Positive | Negative | Total |  |  |  |  |
| CAPI-PCR | Positive | 3 | 0 | 3 |  | Sensitivity (95% CI) | 0,75 | (0,19-0,99) |
|  | Negative | 1 | 116 | 117 |  | Spesificity (95% CI) | 1,00 | (0,97-1,00) |
|  | Total | 4 | 116 | 120 |  |  |  |  |
